# Interdisciplinary research is the key

**DOI:** 10.3389/fnhum.2013.00562

**Published:** 2013-09-13

**Authors:** David A. Waldman

**Affiliations:** Department of Management, W. P. Carey School of Business, Arizona State UniversityTempe, AZ, USA

**Keywords:** neurosciences, organizational neuroscience, organizational behavior, organizational sciences, interdisciplinary communication

The organizational sciences are rapidly coming together with neuroscience theory and methods to provide new insights into organizational phenomena (Becker et al., 2011; Senior et al., 2011; Lee et al., 2012), and even the potential development of individuals within organizations (Waldman et al., 2011). A number of challenges become relevant in the pursuit of such an amalgamation, but perhaps the most apparent is the inherent need for interdisciplinary perspectives and research. An overall purpose of this opinion piece is to clarify the importance of interdisciplinary efforts, while at the same time clarifying the challenges to be faced if we are to apply neuroscience to organizations.

Scientists are typically trained and reinforced to work in a unidisciplinary, specialized mode. It really does not matter if we are considering people trained in the so-called “soft” sciences (e.g., psychology), or whether they come from the “hard” sciences (e.g., neuroscience). We are largely groomed and later reinforced to be specialists. I personally was trained in industrial/organizational psychology, a specialized area of the broader field of psychology. When I was undergoing my graduate education, as well as in the years that followed, I never dreamed that I would someday be working with neuroscientists. But it is now happening. In other words, I am conducting interdisciplinary research involving neuroscientists. In so doing, I certainly do not represent the norm among my colleagues. I say this as a professor in a management department of business school. I realize that for many academic psychologists working in psychology departments, the notion of combining psychology and neuroscience has become the norm. Accordingly, much of what I will address in this opinion piece would not apply to them.

I will address three primary questions in this article. First, what are the institutional and personal impediments that may prevent researchers, especially those in settings such as my own, from engaging in the type of interdisciplinary research that might involve neuroscience? Second, what is the myth vs. reality of the obstacles that might preclude the success of interdisciplinary efforts? Third, what steps can we take to engage in more interdisciplinary research? By addressing these questions, I hope to provide some insight into the issues and benefits of an interdisciplinary approach to neuroscience research. Most of my approach is framed through the perspective of an organizational researcher such as myself, although I conclude with some consideration of why neuroscientists might want to pursue interdisciplinary research that reaches out to the organizational sciences.

## Institutional and personal impediments

I first attempted to apply neuroscience to my own area of specialized expertise, leadership in organizations, around 2005. Early on, I made a presentation on the subject and described some recent data collection efforts to my colleagues at Arizona State University. After the presentation was over, one of my colleagues took me aside and said that what I was attempting to do was quite interesting. He also acknowledged that he had never conceived of such possibilities, largely because of the institutional context in which we exist (about which I will say more below). A second colleague who pulled me aside was more cautionary. He essentially acknowledged that what I was doing was innovative, but recommended, “don't quit your day job.” In other words, the not-so-subtle message was that such interdisciplinary efforts would not end up being rewarded, and I should just stick with the tried and true of unidisciplinary or specialized research activities. Was he correct?

Before answering that question, let's consider how interdisciplinary research can exist at different levels or degrees. As a management professor specializing in micro-level, organizational behavior, let's assume that I want to be more interdisciplinary in my work. I could potentially work on research projects that integrate more macro-level phenomena. Indeed, over the past 20 years I have written on such topics as strategic leadership (e.g., Waldman et al., 2001), corporate social responsibility (e.g., Waldman et al., 2006), and university technology transfer (Siegel et al., 2003). My interdisciplinary work in these areas has brought me together with strategic management and information systems researchers, economists, and financial researchers. The common denominator, however, is that all of this work, and the individuals associated with it, can be placed under the broad umbrella of business-based research. By engaging in interdisciplinary research involving neuroscience, one is “taking a walk on the wild side,” so to speak, and perhaps this is what my colleague was thinking about when he cautioned me to “don't quit your day job.”

So what exactly are the institutional impediments all about? Many of us conduct our research within the institutional confines of universities and research outlets, specifically journals. Historically, the structure of universities is very segmented or siloed. Even the physical buildings in which our offices are housed tend to maintain this segmentation, e.g., offices for people in a particular department or disciplinary area are largely in the same location. Perhaps more importantly, our reward systems (e.g., promotion and tenure) tend to reinforce specialization. As an organizational researcher, I have received messages (some subtle, some not so subtle) throughout my career that while some dabbling in other areas might be permissible, I should not stray too far or too much from my own specialization, or else my own tenure, promotion, and reputation could be put at risk. Moreover and relatedly, I have been told that the best journals will not accept highly interdisciplinary research. Below I will attempt to separate the myth from reality with regard to publication issues.

Most of us are keenly aware of the structural or institutional impediments to interdisciplinary research. But perhaps we are not so cognizant of our own personal issues that might preclude us from engaging in such research. We are conditioned early on as graduate students to work on specialized projects. After graduation, we are then encouraged to gradually make a name for ourselves in particular, focused streams of research. Rarely does the thought of interdisciplinary activities take hold. Indeed, the networks that we form, conferences that we attend, and so forth, center around unidisciplinary work. In short, we can get by just fine in our careers without becoming interdisciplinary. So why bother?

## Separating myth from reality

Before I provide my take on this question, I first want to separate some myth from reality. The first myth is that researchers from widely disparate disciplines either cannot, or will not, come together to pursue interdisciplinary efforts. As an organizational behaviorist, I will admit to having mixed luck with regard to collaborative relationships with neuroscientists. At times, it has been challenging because of differing goals, perspectives, and the reality that some neuroscientists themselves may not be interested in the pursuit of interdisciplinary research.

But for the most part, I have been able to form beneficial connections with such individuals, and together we have attempted to dispel a second myth. Specifically, there is the myth that top journals in organizational/management will not accept interdisciplinary research, especially when it crosses such a seemingly huge boundary as the neuroscience realm. This myth personifies the fear that my colleague mentioned back in 2005 when he cautioned me to not quit my day job. The fear was that I simply would not be able to place such research in the top journals in my field. To be sure, at the time, there were no neuroscience-based articles in organizational/management journals. So his conclusion might seem warranted. In addition, interdisciplinary submissions can create difficulties for journal editors, for example, finding suitable reviewers. However, the more entrepreneurially-oriented editors of journals in my field increasingly see the potential value in accepting at least some interdisciplinary articles, including those involving neuroscience concepts and methods. In speaking with editors of journals in my field, they seem keenly aware of how neuroscience is affecting other fields in business. Examples include neuro-economics (e.g., Braeutigam, 2005; Camerer et al., 2005; Kenning and Plassman, 2005) and neuro-marketing (e.g., Lee et al., 2007). So inclusion of neuroscience-based articles is rapidly being viewed as more normal, and less revolutionary. Since 2005, I personally have been able to achieve a least a modicum of success in such publication efforts, largely involving neuroscientists as co-authors (Peterson et al., 2008; Balthazard et al., 2012; Hannah et al., 2013; Waldman et al., 2013). Moreover, it is my experience that grant agencies and foundations increasingly seek interdisciplinary research proposals that involve co-investigators from diverse backgrounds.

## Steps toward becoming more interdisciplinary

The type of interdisciplinary research that I have described here can be framed in terms of the classic approach-avoidance conflict. To a large extent, I have emphasized the salience of the approach aspects that might make a researcher want to proceed with interdisciplinary work, while minimizing potential avoidance reasons for shunning pursuits of this nature. With that said, I fully realize that a key consideration on the avoidance side is the ambiguity inherent in determining when or how to make it happen. In other words, when and how might one become more interdisciplinary in his/her approach to research, especially with regard to combining neuroscience with fields of study such as the organizational sciences? For individuals whose primary focus is the latter, the first thing that I would caution is to treat the potential integration of neuroscience as more of a personal vision, rather than predominant reality, early on in one's career. In other words, as a doctoral student and in the early portion of one's career, it might be best to focus largely on developing a focused specialization, while at the same time keeping in mind and gradually working toward interdisciplinary possibilities.

Once one has determined to become more interdisciplinary, there are two avenues that might be pursued. First, an individual can simply expand his or her own domain of expertise to include an area such as neuroscience. The obvious limitation of this approach is that we all have time constraints, as well as demands to maintain expertise in our own specialized areas. To some degree, I personally have followed this route. But because of the sheer breadth and complexity of neuroscience, I have chosen a second avenue for approaching neuroscience. Specifically, I have partnered with trained neuroscientists in terms of both publication and grant activities. Indeed, I have found this second avenue to be especially important as a means of providing a better perspective of neuroscience, and to deal with the complexities of actual data collection and analysis processes (e.g., Balthazard et al., 2012). For example, through collaboration with neuroscientists, I have gained a better feel for what “activity” in brain regions may operationally be all about, as well as the potential relevance of both intrinsic and reflexive brain activity to organizational phenomena (Waldman et al., 2013).

## Concluding thoughts

Throughout this opinion piece, I have focused on interdisciplinary work from the viewpoint of a non-neuroscientist, such as myself. But what about neuroscientists; what might be their motivation to work with organizational researchers? In my own experience, I have had much more success at connecting with neuroscientists who combine the scientist-practitioner model, including establishing their own firms to produce applications to such maladies as attention deficit disorder, sleep apnea, and so forth. These individuals have bonafide credentials in terms of their basic understanding of neuroscience theory and methods, but they are also interested in real-world applications. Thus, it is a natural extension of their work to look toward the organizational world to see how their expertise might be applied. In contrast, I have had less luck connecting with “pure” academics, for example, social cognitive neuroscientists who might be working in psychology departments of universities. However, I recognize that there will be more such connections between organizational researchers and basic neuroscience researchers in the future.

In conclusion, it is my hope that this commentary will help to provide some insights into the issues and advantages pertaining to interdisciplinary research in the realm of organizations and neuroscience. There is much potential for research of this nature to address some of the larger problems facing organizations. In turn, by focusing attention on organizational issues, new insights and opportunities may present themselves for neuroscientists.

## References

[B1] Balthazard P. Waldman D. A. Thatcher R. W. Hannah S. T. (2012). Differentiating transformational and non-transformational leaders on the basis of neurological imaging. Leadership Quart. 23, 244–258 10.1016/j.leaqua.2011.08.002

[B2] Becker W. J. Cropanzano R. Sanfey A. G. (2011). Organizational neuroscience: taking organizational theory inside the neural black box. J. Manage. 37, 933–961 10.1177/0149206311398955

[B3] Braeutigam S. (2005). Neuroeconomics—from neural systems to economic behaviour. Brain Res. Bull. 67, 355–360 10.1016/j.brainresbull.2005.06.009 16216681

[B4] Camerer C. Loewenstein G. Prelec D. (2005). Neuroeconomics: how neuroscience can inform economics. J. Econ. Lit. 43, 9–64 10.1257/0022051053737843

[B5] Hannah S. T. Balthazard P. A. Waldman D. A. Jennings P. Thatcher R. (2013). The psychological and neurological bases of leader self-complexity and effects on adaptive decision-making. J. Appl. Psychol. 98, 393–411 10.1037/a0032257 23544481

[B6] Kenning P. Plassman H. (2005). NeuroEconomics: an overview from an economic perspective. Brain Res. Bull. 67, 343–354 10.1016/j.brainresbull.2005.07.006 16216680

[B7] Lee N. J. Senior C. Butler M. J. R. (2012). The domain of organizational cognitive neuroscience: theoretical and empirical challenges. J. Manage. 38, 921–931 10.1177/0149206312439471

[B8] Lee N. Broderick A. J. Chamberlain L. (2007). What is ‘neuromarketing’? A discussion and agenda for future research. Int. J. Psychophysiol. 63, 199–204 10.1016/j.ijpsycho.2006.03.007 16769143

[B9] Peterson S. Balthazard P. A. Waldman D. A. Thatcher R. W. (2008). Neuroscientific implications of psychological capital: are the brains of optimistic, hopeful, confident, and resilient leaders different? Organ. Dyn. 37, 342–353 10.1016/j.ougdyn.2008.07.007

[B10] Senior C. Lee N. J. Butler M. J. R. (2011). Organizational cognitive neuroscience. Organ. Sci. 22, 804–815 10.1287/orsc.1100.0532

[B11] Siegel D. Waldman D. A. Link A. (2003). Assessing the impact of organizational practices on the relative productivity of university technology transfer offices: an exploratory study. Res. Policy 32, 27–48 10.1016/S0048-7333(01)00196-2

[B12] Waldman D. A. Balthazard P. A. Peterson S. (2011). The neuroscience of leadership: can we revolutionize the way that leaders are identified and developed? Acad. Manage. Perspect. 25, 60–74 10.5465/AMP.2011.59198450

[B13] Waldman D. A. Ramírez G. House R. J. Puranam P. (2001). Does leadership matter? CEO leadership attributes and profitability under conditions of perceived environmental uncertainty. Acad. Manage. J. 44, 134–144 10.2307/3069341

[B14] Waldman D. A. Siegel D. Javidan M. (2006). Components of transformational leadership and corporate social responsibility. J. Manage. Stud. 43, 1703–1725 10.1111/j.1467-6486.2006.00642.x

[B15] Waldman D. A. Wang D. Berka C. Stikic M. Balthazard P. A. Richardson T. (2013). Emergent leadership and team engagement: an application of neuroscience technology and methods, in Paper presented at the Meeting of the Academy of Management, Orlando, August, and published in the Best Paper Proceedings, (Orlando, FL)

